# In Silico Pan-Cancer Analysis Reveals Prognostic Role of the Erythroferrone (ERFE) Gene in Human Malignancies

**DOI:** 10.3390/ijms24021725

**Published:** 2023-01-15

**Authors:** Qingyu Xu, Eva Altrock, Nanni Schmitt, Alexander Streuer, Felicitas Rapp, Verena Nowak, Julia Obländer, Nadine Weimer, Iris Palme, Melda Göl, Wolf-Karsten Hofmann, Daniel Nowak, Vladimir Riabov

**Affiliations:** Department of Hematology and Oncology, Medical Faculty Mannheim, Heidelberg University, 68169 Mannheim, Germany

**Keywords:** ERFE, pan-cancer, prognostic biomarker, tumor microenvironment, NOTCH, WNT, PI3K-AKT, tumorigenesis, metastasis

## Abstract

The erythroferrone gene (*ERFE*), also termed *CTRP15*, belongs to the C1q tumor necrosis factor-related protein (CTRP) family. Despite multiple reports about the involvement of CTRPs in cancer, the role of ERFE in cancer progression is largely unknown. We previously found that *ERFE* was upregulated in erythroid progenitors in myelodysplastic syndromes and strongly predicted overall survival. To understand the potential molecular interactions and identify cues for further functional investigation and the prognostic impact of ERFE in other malignancies, we performed a pan-cancer in silico analysis utilizing the Cancer Genome Atlas datasets. Our analysis shows that the *ERFE* mRNA is significantly overexpressed in 22 tumors and affects the prognosis in 11 cancer types. In certain tumors such as breast cancer and adrenocortical carcinoma, *ERFE* overexpression has been associated with the presence of oncogenic mutations and a higher tumor mutational burden. The expression of *ERFE* is co-regulated with the factors and pathways involved in cancer progression and metastasis, including activated pathways of the cell cycle, extracellular matrix/tumor microenvironment, G protein-coupled receptor, NOTCH, WNT, and PI3 kinase-AKT. Moreover, *ERFE* expression influences intratumoral immune cell infiltration. Conclusively, *ERFE* is aberrantly expressed in pan-cancer and can potentially function as a prognostic biomarker based on its putative functions during tumorigenesis and tumor development.

## 1. Introduction

C1q tumor necrosis factor (TNF)-related proteins (CTRPs) belong to the adipokine superfamily and comprise 15 members in addition to adiponectin (CTRP1-CTRP15) [1]. CTRPs are involved in the regulation of numerous physiological and pathological processes, such as cell proliferation, inflammation, apoptosis, glycolipid metabolism, and protein kinase pathways [2]. 

Due to these functions, CTRPs also play crucial roles in the development and progression of various cancer types [2]. In particular, CTRP1, CTRP3, CTRP4, CTRP6, and CTRP8 are frequently reported to be involved in carcinogenesis. Multiple studies have reported pro-tumor functions of these CTRPs in cancer, which are primarily attributed to their stimulating effects on tumor cell survival, proliferation, invasion, and angiogenesis [2,3,4,5,6,7]. These tumor-supportive functions have been associated with the activation of various signaling cascades known to play a role in cancer progression, including extracellular signal-regulated protein kinases 1 and 2 (ERK1/2), mitogen-activated protein kinase/ERK1/2, and PI3-kinase (PI3K)/AKT pathways [2,8,9]. The activation of these pathways results in an increased production of pro-inflammatory mediators, activation of the cell cycle, and inhibition of apoptosis [2,3,4,5,6,7,8,9]. Because of the important roles of CTRPs in tumorigenesis, CTRPs could possibly be considered as diagnostic and prognostic biomarkers or therapeutic targets. Due to the recently discovered prognostic and functional role in iron homeostasis in myeloid neoplasia [10,11], in this study we focused on the somatic expression profiles and putative cancer-related functions of CTRP15 (also named as erythroferrone [ERFE] and myonectin). 

ERFE is a multifaceted protein that has been shown to function as an adipokine, myokine, hormone, and inflammatory regulator depending on the tissue context and pathology [12,13,14]. Its unique function is a crucial involvement in the regulation of systemic iron metabolism, which is severely disrupted in myeloid neoplasia and commonly altered in other cancer types [10]. Moreover, similar to other CTRPs, ERFE takes part in lipid metabolism, increasing fatty acid uptake in adipocytes and the expression of genes associated with fatty acid binding and transport, such as *CD36*, *FABP4*, and *FATP1* [15]. There is abundant evidence that adipocytes are a crucial part of the tumor microenvironment (TME), and dysregulated lipid metabolism is one of the most prominent metabolic alterations in tumors [16] since tumor cells utilize altered lipid metabolism to synthesize the molecules responsible for cell proliferation, survival, invasion, and metastasis. Altogether, these data suggest that ERFE might play a role in tumorigenesis and potentially affect prognosis in patients with cancers. However, a comprehensive assessment of *ERFE* expression in cancerous tissues and its association with cancer has not been performed yet. In this study, we carried out a comprehensive in silico analysis for the *ERFE* gene based on publicly available Omics data to further investigate the potential molecular mechanisms by which *ERFE* contributes to tumorigenesis and prognosis in cancer. Through detailed analyses of mRNA expression and its associations with prognosis, mutational burden, immune infiltrates, and the enrichment of signaling pathways, the role of the *ERFE* gene in 33 types of cancer was evaluated.

## 2. Results

### 2.1. ERFE Is Aberrantly Expressed in Cancer Tissues 

**Figure 1:**
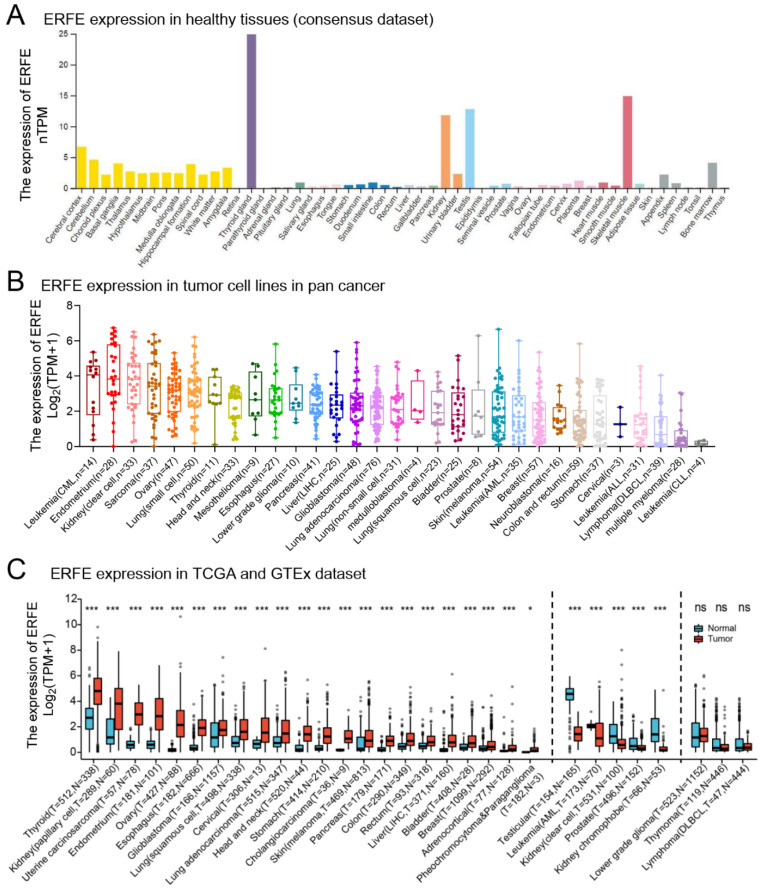
Expression levels of the *ERFE* gene in human normal tissues and pan-cancer. (**A**) Consensus *ERFE* healthy tissue expression based on datasets of HPA, GTEx, and FANTOM5 (function annotation of the mammalian genome). (**B**) The expression distribution of the *ERFE* gene was visualized in 31 cancer types containing 1018 tumor cell lines from the CCLE dataset. (**C**) The expression distribution of the *ERFE* gene was visualized between the investigated 33 cancer types from the TCGA project and normal tissues from the GTEx database. The expression difference between the tumor and healthy groups was compared using the Wilcoxon rank sum test. Asterisks (*) stand for significance levels. ns, *p* ≥ 0.05; * *p* < 0.05; *** *p* < 0.001. Abbreviations: PCPG, pheochromocytoma and paraganglioma; T, tumor; N, normal tissues; CCLE, Cancer Cell Line Encyclopedia dataset; FANTOM5, Functional ANnoTation Of the Mammalian genome project 5.

We first studied the mRNA expression level of *ERFE* in various healthy human tissues using the Human Protein Atlas dataset (HPA, https://www.proteinatlas.org/ (accessed on 13 July 2022)). We found that *ERFE* was most strongly expressed in thyroid tissue, followed by skeletal muscle, testis, kidney, brain (e.g., cerebral cortex and cerebellum), bone marrow, urinary bladder, and appendix (Figure 1A). 

Although *ERFE* expression was limited to several healthy tissues, we found that *ERFE* was widely expressed in cancer cell lines (Figure 1B). Furthermore, we compared *ERFE* expression between primary bulk tumor tissues and corresponding normal tissues by integrating datasets from the Genotype-Tissue Expression (GTEx) and the Cancer Genome Atlas (TCGA) (Figure 1C). *ERFE* was consistently overexpressed in tumor tissue in comparison to the normal tissue controls in 22 out of 33 tumor types. In n = 5 cancer entities, *ERFE* was significantly downregulated in tumor tissues as compared to the matched healthy tissues (Figure 1C).

In summary, we found that the *ERFE* gene is widely deregulated in tumor tissues as compared to normal controls.

### 2.2. ERFE Expression Is Independently Associated with Survival in Several Cancer Types 

**Figure 2:**
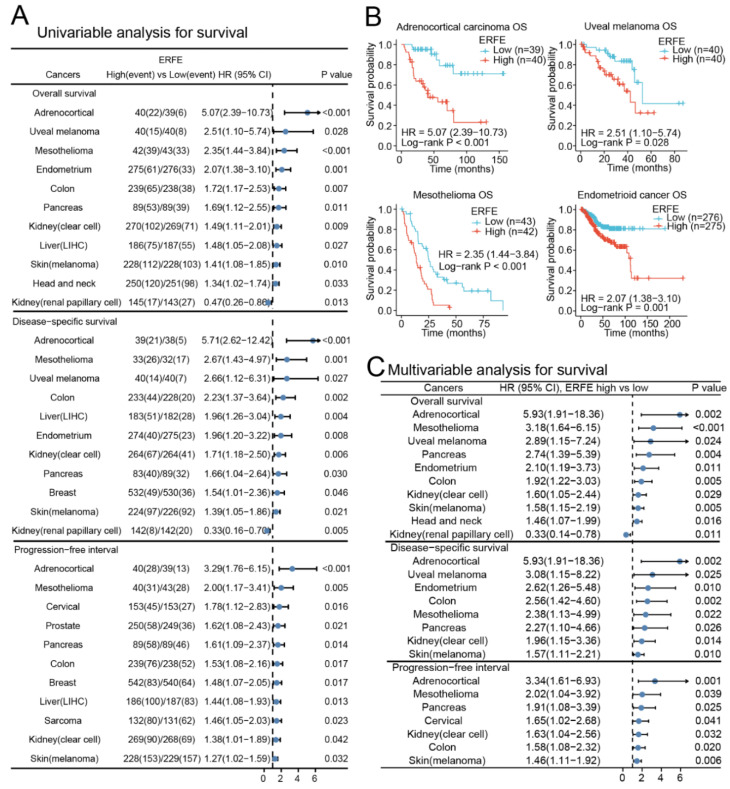
Prognostic significance of *ERFE* expression in pan-cancer. (**A**) Forest plot of survival (OS, DSS, and PFI) associations with *ERFE* expression levels in univariable analyses. Log-rank test was conducted in pan-cancer and results with *p* < 0.05 were summarized. (**B**) Examples of survival analysis are shown. Kaplan–Meier analysis was performed. (**C**) Forest plot of OS, DSS, and PFI associations with *ERFE* expression levels in multivariable analyses. Cox regression analysis was conducted using TNM and treatments as confounders. Results with *p* < 0.05 were summarized.

Next, we assessed whether deregulated expression of *ERFE* is of prognostic significance in pan-cancer. We grouped patients into *ERFE^high^* and *ERFE^low^* groups based on the median expression in each tumor. In a univariable analysis, *ERFE* expression was significantly associated with inferior overall survival (OS) and disease-specific survival (DSS) in n = 10 tumor types as well as inferior progression-free interval (PFI) in n = 11 tumor types (Figure 2A). Among the analyzed tumors, the strongest associations with OS were observed in adrenocortical carcinoma, uveal melanoma, mesothelioma, and endometrioid cancer (Figure 2B). We further validated the associations of *ERFE* expression with survival in n = 11 tumor types using Cox regression models that adjusted survival data for clinical tumor (TNM) stages and treatments (Figure 2C and Table S1). The multivariable analysis confirmed that the high *ERFE* expression was independently associated with inferior OS in n = 10 tumor types, inferior DSS in n = 8 tumor types, and inferior PFI in n = 7 tumor types. Higher *ERFE* expression was related to all three types of survival (OS, DSS, and PFI) in adrenocortical cancer, mesothelioma, pancreatic, colon, kidney clear cell, and skin cutaneous melanoma cancers, and indicated poor outcome. Only in kidney renal papillary cell carcinoma, *ERFE* overexpression correlated with superior OS (Figure 2C, Table S1). 

Overall, high *ERFE* expression was related to inferior prognosis in most analyzed cancer entities.

### 2.3. Mutational Frequencies and Tumor Mutational Burden (TMB) Are Associated with ERFE Expression Levels

**Figure 3:**
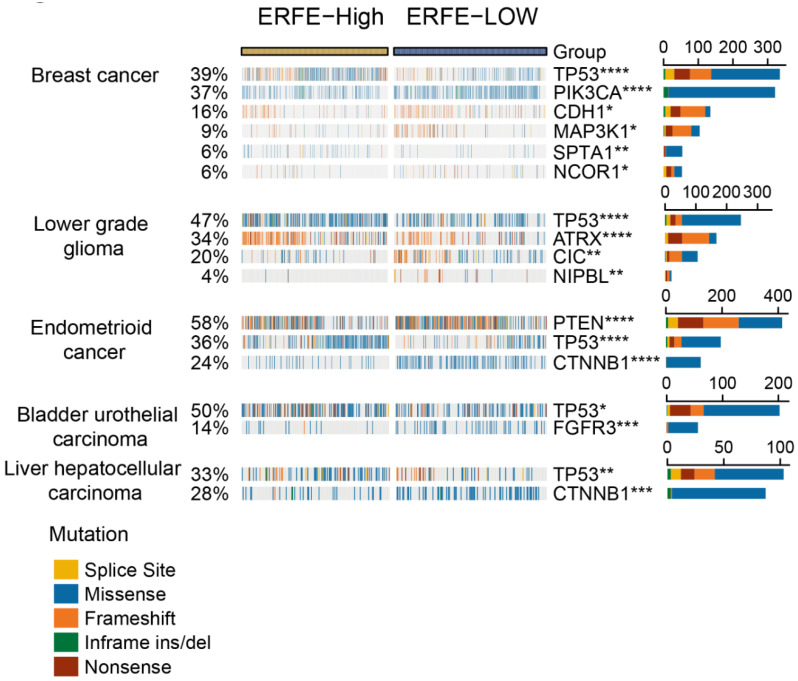
Correlation of *ERFE* expression status with genetic alterations. An oncoplot is presented for the top 20 frequently mutated genes significantly correlated with *ERFE* expression levels in pan-cancer. Fisher’s exact test was conducted and results with adjusted *p* < 0.05 are displayed. * *p* < 0.05; ** *p* < 0.01; *** *p* < 0.001; **** *p* < 0.0001.

Due to the strong association of the *ERFE* expression with prognosis in multiple types of cancer, we next sought to provide explanations for this observation via analysis of available mutational data. Using the website tool of Comprehensive Analysis on Multi-Omics of Immunotherapy in pan-cancer (CAMOIP) [17], we found that the frequencies of mutations in several tumor suppressor genes (e.g., *TP53* and *PTEN*) and oncogenes (e.g., *CTNNB1*) were unequally distributed in the *ERFE^high^* versus *ERFE^low^* groups. Of note, there was a strong association of *ERFE* overexpression with a higher frequency of *TP53* mutations in breast, endometrioid, bladder, and liver cancers, and lower-grade glioma (Figure 3). 

**Figure 4:**
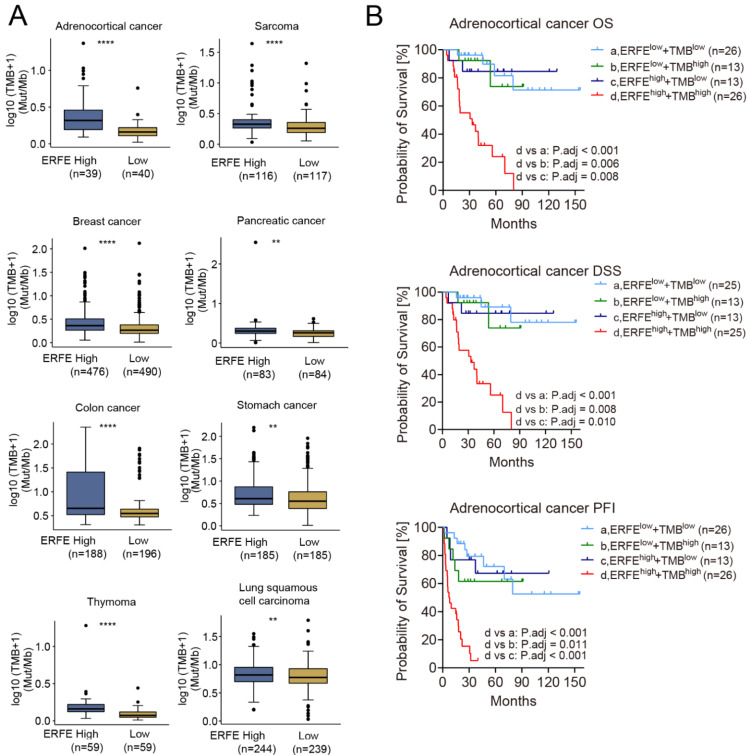
Correlation of *ERFE* expression status with TMB. (**A**) Comparison of TMB between *ERFE^high^* and *ERFE^low^* patients in pan-cancer. Wilcoxon rank sum test was performed. ** *p* < 0.01; **** *p* < 0.0001. (**B**) Patients with different levels of *ERFE* expression and TMB were stratified into groups of *ERFE^low^*TMB^low^, *ERFE^low^*TMB^high^, *ERFE^high^*TMB^low^, and *ERFE^high^*TMB^high^ in adrenocortical cancer. The survival subgroup analysis was analyzed by Log-rank test with multiple comparisons for calculating adjusted *p*-values.

Due to the difference in the frequency of mutations in the *ERFE^high^* and *ERFE^low^* groups and the unequal distribution of *TP53* mutations, an important driver of genomic instability, we compared the TMB between the two groups. Interestingly, the TMB was significantly higher in the *ERFE^high^* groups in n = 8 tumor types (Figure 4A). Among them, *ERFE* overexpression indicated poor prognosis in adrenocortical, pancreatic, and colon cancers (Figure 2C). Remarkably, in adrenocortical cancer, a shorter OS, DSS, and PFI due to the TMB^high^ status was offset in the *ERFE^low^* patients, whereas the *ERFE^high^*TMB^high^ status was a very strong indicator of poor OS, DSS, and PFI in this tumor (Figure S1 and Figure 4B). Overall, our data shows an association between *ERFE* expression and TMB as well as a potential functional interplay between these two factors, which may be relevant for patient survival. 

### 2.4. ERFE^high^ Status Is Associated with Activated Cell Cycle 

**Figure 5:**
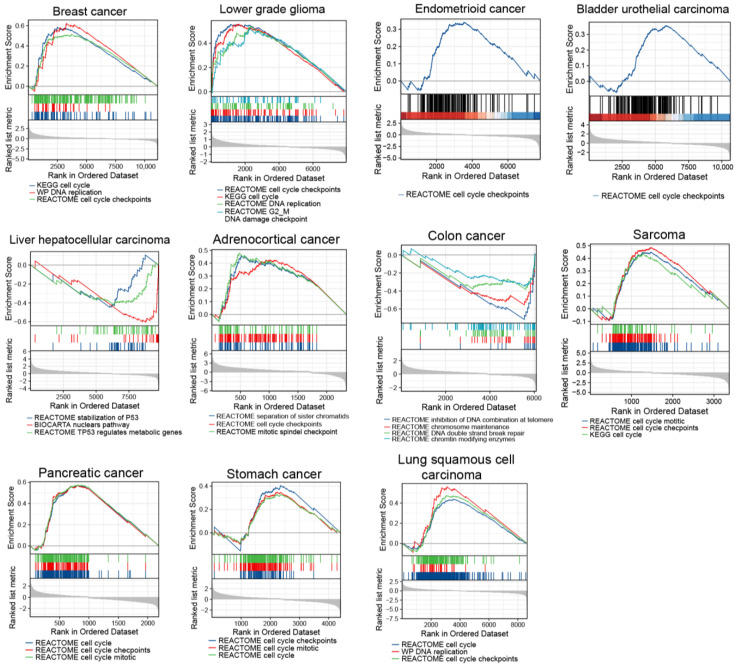
GSEA results are shown for upregulated pathways involved in cell cycle and DNA replication as well as inhibited pathways involved in stabilization of P53 and chromosomal maintenance.

Since *TP53* mutations and higher TMB are associated with genomic instability, which contribute to carcinogenesis and tumor cell proliferation [18,19], we assessed cell cycle states in *ERFE^high^* cancer samples. Therefore, we carried out a single-gene differential analysis (SGDA) followed by a gene set enrichment analysis (GSEA) based on the identified differentially expressed genes (DEGs) between the *ERFE^high^* and *ERFE^low^* groups in the tumors shown in Figure 3 and Figure 4A. Interestingly, the activated pathways involved in cell cycle and DNA replication as well as the processes involved in P53 stabilization and chromosomal maintenance were enriched in these tumors (Figure 5).

### 2.5. The Genes with Tumor-Supportive Functions Are Strongly Co-Expressed with ERFE 

**Figure 6:**
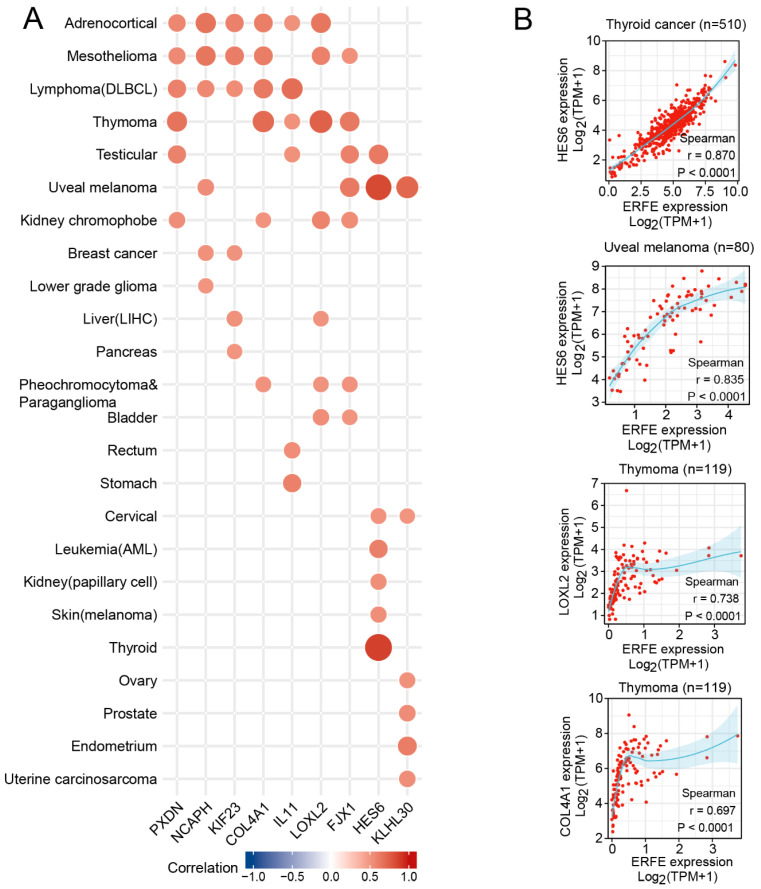
Expression correlation between *ERFE* and other functional genes. Spearman’s rank correlation test was carried out for each tumor type. (**A**) Genes with Spearman r threshold >0.5 and *p* < 0.0001 were listed when covering at least six tumor types. (**B**) Examples of genes with Spearman r threshold >0.6 and *p* < 0.0001 were shown in thyroid cancer, uveal melanoma, and thymoma.

We next assessed which genes crucially involved in carcinogenesis are significantly co-expressed with *ERFE*. We first identified genes that were strongly and positively co-regulated with *ERFE* at the mRNA expression level (Spearman r > 0.5, *p* < 0.0001) in at least six tumor types. This analysis identified nine genes with known involvement in cancer pathogenesis (Figure 6). Among them, the *HES6* gene is a component of activated NOTCH signaling [20,21], whereas *KIF23* and *NCAPH* support cell cycle progression via facilitating cytokinesis during mitosis and separation of chromosomes, respectively [22,23,24,25], and *KIF23* also promotes WNT signaling [26]. 

**Figure 7:**
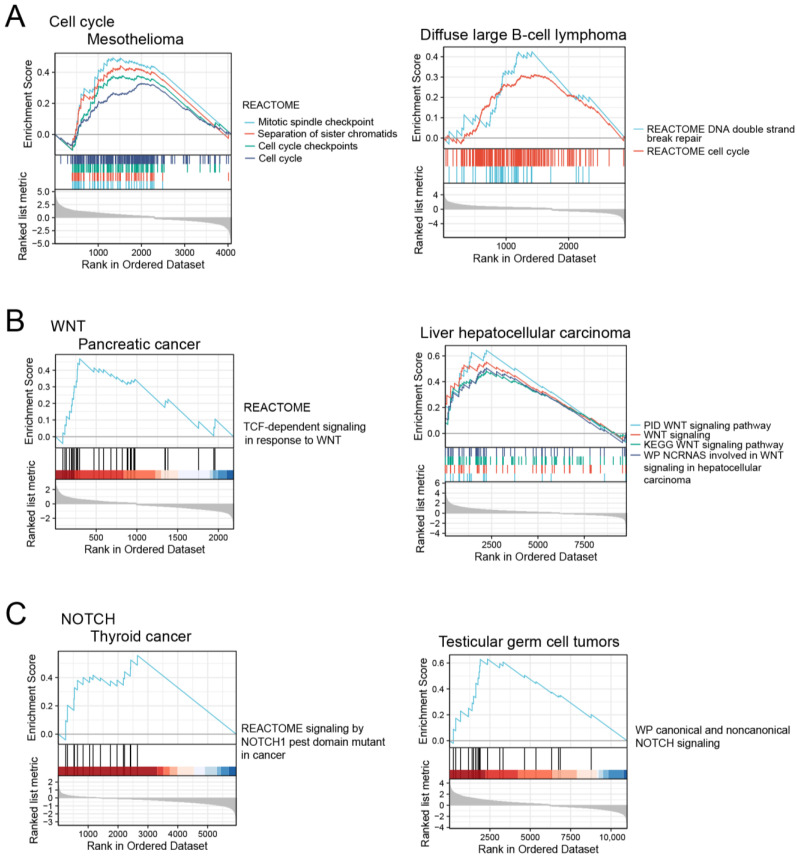
The activation of cell cycle (**A**), WNT (**B**), and NOTCH signaling pathways (**C**) in *ERFE^high^* tumor samples based on GSEA analysis.

Indeed, the GSEA analysis showed that the corresponding pathways were enriched in *ERFE^high^* tumor samples (Figure 7). In addition, *ERFE* was co-expressed with the genes involved in extracellular matrix (ECM) deposition and remodeling, including *PXDN*, *COL4A1*, and *LOXL2* that are known to promote cancer invasion and metastasis [27,28,29,30]. Of note, the highest number of these genes (n = 6) was positively co-regulated with *ERFE* in adrenocortical cancer and mesothelioma, cancers where high *ERFE* expression showed the strongest association with inferior survival (Figure 2C).

### 2.6. ERFE^high^ Status Correlates with the Changes in the Tumor Microenvironment and Activation of Tumor-Supportive Signaling Pathways 

**Figure 8:**
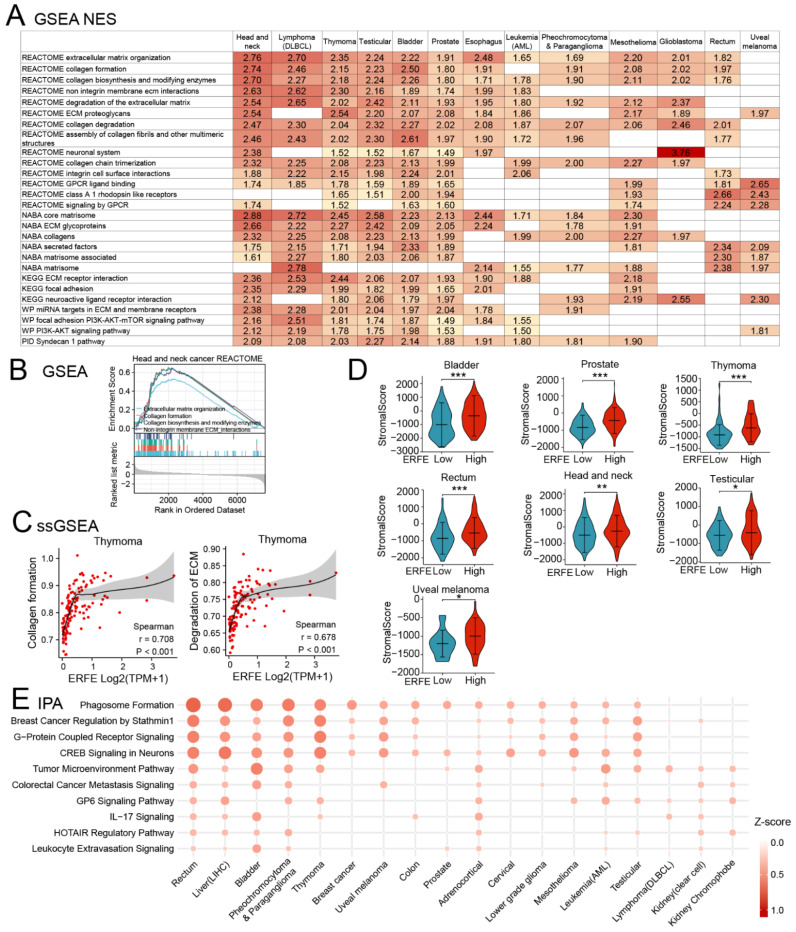
Upregulated pathways involved in higher *ERFE* expression. (**A**) Upregulated pathways upon *ERFE^high^* status were enriched by GSEA based on REACTOME, NABA, KEGG, WP, and PID databases and summarized with NES ≥ 1 and *p* < 0.05. (**B**) Representative GSEA result is shown for upregulated pathway involved in ECM remodeling. (**C**) Correlation between *ERFE* expression and pathway scores of “Collagen formation” and “Degradation of ECM” in thymoma is shown. Pathway score was calculated by ssGSEA. Spearman’s rank correlation test was carried out. (**D**) Stroma infiltration abundance was compared between *ERFE^high^* and *ERFE^low^* patients in pan-cancer. Stroma score was calculated by the ESTIMATE algorithm. The statistical difference of the two groups was compared by Wilcoxon rank sum test. Asterisks (*) stand for significance levels. * *p* < 0.05; ** *p* < 0.01; *** *p* < 0.001. (**E**) Upregulated pathways upon *ERFE^high^* status were enriched by IPA and summarized with Z-score ≥ 1 and *p* < 0.05. Abbreviation: NES, normalized enrichment score.

We further comprehensively characterized the functional pathways enriched in *ERFE^high^* tumors using GSEA in pan-cancer. To increase the validity of the findings, each *ERFE^high^*-associated pathway had to be enriched in at least nine tumor types. GSEA and single-sample GSEA (ssGSEA) analyses identified the enrichment of multiple pathways associated with ECM formation, organization, and processing as well as cell-to-ECM interaction (Figure 8A–C). These results were in line with our data on the positive co-regulation of *ERFE* with the genes (*LOXL2*, *PXDN* and *COL4A1*) involved in ECM deposition and processing (Figure 6A). Interestingly, in 7 out of 13 tumor types shown in Figure 8A, the enrichment of ECM pathways in the *ERFE^high^* group was linked to an increased abundance of stromal infiltrate (stromal score), which could be responsible for the increased deposition of ECM (Figure 8D). In addition, GSEA revealed that the *ERFE^high^* status was associated with the enrichment of the G protein coupled receptor (GPCR) and PI3K-AKT pathways that are frequently overactivated in cancers and support tumor cell survival, proliferation, and invasion [31,32,33]. 

We additionally utilized the Ingenuity Pathway Analysis (IPA) for an independent pathway enrichment analysis (Figure 8E). The IPA confirmed the enrichment of pathways involved in TME and ECM, which are reported to be essential non-cellular components of TME [34], GPCR, and PI3K activation (as indicated by breast cancer regulation by Stathmin 1 pathway in IPA [35]) in *ERFE^high^* cancers (Figure 8E and Figures S2–S5). The ECM remodeling and the activation of these signaling pathways are crucial for distant metastases [28]. Indeed, our analysis revealed that *ERFE* overexpression was pronouncedly associated with the presence of metastases in prostate cancer and melanoma (Figure S6).

In addition to the enrichment in the TME pathway, the IPA also showed an association of the interleukin-17 (IL-17) signaling pathway with the *ERFE^high^* status (Figure 8E). A more detailed pathway enrichment analysis using the IPA suggested that the differentiation/recruitment of the T helper 2 (Th2) and Th17 cells, the production of multiple chemokines, and the pro-inflammatory mediators could be affected by the *ERFE^high^* status (Figure S7). Therefore, we performed the analysis of Th2 and Th17 infiltration using ssGSEA algorithms. Indeed, the results showed significant positive associations between Th2 cell infiltration and *ERFE^high^* status in multiple cancers, which were especially pronounced in mesothelioma and adrenocortical cancer (Figure S8). 

Single cell RNA sequencing datasets available at “scTIME Portal” [36] showed that the *ERFE* gene was not expressed in a broad range of immune cells (e.g., T cells, B cells, NK cells, macrophages, monocytes, neutrophils, etc.) in pan-cancer (data not shown). Therefore, we further comprehensively assessed the association of multiple immune cell infiltration with *ERFE* expression in tumor cells. An unsupervised consensus clustering identified three distinct clusters with clusters 1 and 2 displaying a negative correlation between immune cell infiltration and *ERFE* expression, especially for cluster 1 (Figure S9). Of note, inside cluster 1, a high *ERFE* expression was associated with reduced infiltration by CD8+ cytotoxic T cells and antigen presenting cells (dendritic cells). Moreover, *ERFE* expression was negatively associated with the expression of immune checkpoints, including *PDCD1* (encoding PD-1 protein), *CD274* (encoding PD-L1 protein), and *CTLA-4* in testicular cancer and thymoma tumors assigned to cluster 1 (Figure S10A). Since immunosuppression in the TME was frequently reported to be induced by B7/CTLA-4 and PD-1/PD-L1 interactions [37,38,39], we firstly used the TIDE algorithm to predict the response to immune checkpoint blockade (ICB) therapy, including CTLA-4 and PD-1, in testicular cancer and thymoma [40]. Interestingly, an impaired response to ICB therapy was predicted in patients with *ERFE^high^* status (*p* < 0.001, Figure S10B). We further attempted to correlate *ERFE* expression with ICB response in real world clinical settings. The ICB treatment data and associated mRNA expression datasets were available for melanoma patients from several published reports (Tables S2). However, in these small sample size datasets, *ERFE* expression was not associated with a response to ICB and survival (Table S2 for response rate; Figure S10C for OS and PFI summary) [37,38,39]. 

In summary, pathway enrichment analyses revealed a tight association of *ERFE* overexpression with GPCR-activated pathways, activated PI3K-AKT signaling pathway, as well as changes in TME, including ECM remodeling, inflammation, and immune cell recruitment, the processes that are crucially involved in the progression of multiple cancers.

## 3. Discussion

Although cancer-related functions of some CTRPs have been clarified [2], the role of ERFE during tumorigenesis remains unknown. Therefore, our study utilized the TCGA dataset to comprehensively analyze the functions of *ERFE* (*CTRP15*) gene expression in cancer. 

We found that *ERFE* was overexpressed in 22 types of malignancies, suggesting the role of *ERFE* in tumorigenesis. Furthermore, *ERFE* mRNA overexpression in bulk tumor tissues was an independent factor in predicting inferior survival in 11 tumor types, especially in adrenocortical carcinoma, mesothelioma, and uveal melanoma. Due to the aberrant *ERFE* expression and strong prognostic significance in malignancies, we next interrogated the role of *ERFE* in pathogenesis, disease progression, and metastasis.

Our data showed that *ERFE* overexpression was correlated with a higher frequency of *TP53* mutations in five types of tumors such as breast cancer. The pathway enrichment analyses additionally demonstrated that in these five cancers, *ERFE* overexpression was associated with upregulated signaling pathways involved in the cell cycle, mitotic process, and DNA replication, which are tightly associated with genomic instability [18,19]. The identified connection between *ERFE* expression and *TP53* mutations, known inducers of genomic instability, is of potential clinical significance and deserves further functional analysis [41]. In line with this finding, the genomic instability-related pathway was enriched in the *ERFE^high^* group in tumors with a higher TMB such as adrenocortical carcinoma. Previous studies reported that genomic instability could increase the frequency of mutations and thereby contribute to a higher TMB [42]. Overall, our data suggest that the *ERFE* overexpression might be associated with genomic instability, which is linked to tumorigenesis and disease progression. This might explain the particularly dismal prognosis in *ERFE^high^* + TMB^high^ adrenocortical cancer patients shown in Figure 4B. 

In addition, we observed that in certain tumors, such as adrenocortical carcinoma and mesothelioma, *ERFE* was positively co-expressed with the *NCAPH* and *KIF23* genes, which facilitate the separation of chromosomes and cytokinesis during mitosis, thereby promoting tumor cell proliferation [22,23,24,25]. This suggested a potential role of *ERFE* overexpression in tumor progression, which was supported by the presence of activated pathways involved in cell cycle progression and DNA replication in our study. 

Except for the role of *ERFE* in genomics and chromosomes, our study also identified co-expression of *ERFE* and *IL-11*, a known activator of PI3K-AKT and mTOR signaling pathways [43,44]. In addition, *NCAPH* expression was also reported to accelerate the tumor progression via PI3K-AKT signaling [45,46]. The activation of a core cancer regulating PI3K-AKT pathway was further validated by GSEA analysis in nine types of tumors in this study.

Our study additionally identified that *ERFE* overexpression might be related to activated NOTCH-related signaling pathway, which is proven to be significantly involved in the tumorigenesis and tumor invasion in certain cancers including thyroid cancer [47] and uveal melanoma [48]. Our study showed a strong positive correlation between *ERFE* and *HES6* expression in thyroid cancer, uveal melanoma, and testicular germ cell tumors. GSEA results indicated that the *ERFE^high^* status was associated with activated NOTCH-related signaling in thyroid cancer and testicular germ cell tumors, and *HES6* overexpression was shown to contribute to overactivated NOTCH signaling [20]. In addition, one study based on single cell sequencing reported that *HES6* has critical tumorigenic properties downstream the NOTCH signaling pathway and favors motile phenotype of primary uveal melanoma cells [21]. Interestingly, in our study *HES6* was strongly co-expressed with *ERFE* in uveal melanoma, which was associated with distant metastasis. Overall, one could envision that in thyroid cancer, testicular germ cell tumors and uveal melanoma *ERFE* upregulation contribute to NOTCH signaling and its effects on tumor progression, which needs to be determined in functional studies. 

We finally observed a strong association between the *ERFE^high^* status and enrichment in the TME pathway, demonstrating that *ERFE* is associated with ECM formation and remodeling. A previous study also reported that ERFE regulated the differentiation of osteoblasts and osteoclasts in mouse BM cells [49]. Indeed, our study identified a strong correlation between *ERFE* expression and other genes essential for ECM organization, including *PXDN* [27], *COL4A1* [29], and *LOXL2* [30]. It is widely reviewed that collagen-related signaling activation contributes to tumorigenesis and promotes metastasis [28], indicating a potential role of the *ERFE^high^* status in metastasis. Moreover, the *FJX1* gene was also co-expressed with *ERFE* and overexpression of this gene was reported to promote abnormal endothelial capillary tube formation in the TME [50]. Overall, *ERFE* may play a role in TME via ECM remodeling and angiogenesis.

Except for the proposed functions of *ERFE* in tumorigenesis and metastasis, we further assessed the potential role of *ERFE* in immune cell infiltration. The Th2 cells were widely enriched in the *ERFE^high^* cases in pan-cancer. However, an increased infiltration of *ERFE*-high tumors with Tregs was not observed [51]. Previous studies revealed that Th2 cells initiated antitumor responses by type 2 immunity and directly influenced tumor growth and progression [52]. On the other hand, there is also evidence indicating that Th2 immunity promotes carcinogenesis, cancer progression, and metastasis [52]. Currently, the functional consequences of the association of *ERFE* expression with Th2 cell infiltrate are unclear and require additional experimental studies. Importantly, our analysis revealed that in several tumor types, a high *ERFE* expression is associated with a general reduction in immune cell infiltrate, indicating a possible immunosuppression. Moreover, this effect was predicted to manifest in a reduced sensitivity to ICB therapy. Overall, our data suggest that multiple changes in the tumor microenvironment as well as intrinsic changes in tumor cells might underlie the ERFE-associated effects on tumor progression and patient survival. 

It should be noted that our study does not exclude the possibility that ERFE is not a key factor in the progression of many cancers, but rather a molecule that is passively co-regulated with factors strongly involved in cancer progression. To firmly define the role of ERFE in the processes of cancer cell proliferation, migration and metastatic behavior, cellular systems with a direct overexpression or silencing of *ERFE* have to be established and analyzed in functional assays. Nevertheless, the current lack of functional data does not diminish the value of ERFE as a potential prognostic biomarker in many types of cancer. 

In summary, we reported aberrant expression and prognostic significance of ERFE at the pan-cancer level. We also assessed potential functions of *ERFE* gene expression during tumorigenesis, malignant progression, and metastasis. 

## 4. Materials and Methods

### 4.1. Gene Expression Analysis of ERFE

As a landmark project in cancer genomics, TCGA molecularly characterized over 20,000 primary cancer and matched normal tissues covering 33 types of cancer [53]. The GTEx project collected a large number of RNA sequencing samples and multiple traits from 54 types of human tissues [54]. In our study, both public TCGA and GTEx RNA sequencing data were downloaded using the UCSC Xena platform (https://xenabrowser.net/datapages/ (accessed on 14 June 2022)) [55]. The cell line mRNA expression matrix of tumors was obtained from the Cancer Cell Line Encyclopedia (CCLE) dataset (https://portals.broadinstitute.org/ccle (accessed on 20 July 2022)) [56]. All mRNA expression data were processed uniformly by Toil to get transcripts per million (TPM) [57].

The quantification and comparison were based on Log_2_(TPM + 1). To analyze the correlation of *ERFE* expression with other protein-coding mRNAs, STAT package (v.3.6.3) was utilized in R software (v. 4.0.3, Vienna, Austria). Moreover, we constructed the *ERFE* mRNA expression landscape in human healthy bulk tissues using the HPA database (v.21.1, https://www.proteinatlas.org/ (accessed on 13 July 2022)). The clinical and gene expression datasets for Table S2 were downloaded from the TIDE database [40].

### 4.2. Survival Analysis

Survival status was downloaded from the TCGA dataset [58]. For survival analysis associated with *ERFE* expression and TMB, patients were grouped by median expression in each tumor cohort. Kaplan-Meier (KM) survival analysis was performed by the Log-rank test using R packages of survminer (v.0.4.9, ) and survival (v.3.2.10) or Graphpad Prism (v.8, San Diego, CA, USA), and represented as hazard ratio (HR). HR > 1 indicates an increased risk in the group with mutations. Subgroup analysis of survival was analyzed by the Log-rank test showing adjusted *p*-values of multiple comparisons. OS, DSS, and PFI were analyzed as defined previously [58]. Multivariable analyses combining clinical T stages and therapies were performed using the Cox regression analysis. 

### 4.3. Genetic Alteration Analysis Based on ERFE Expression in Pan-Cancer

Pan-cancer genetic alterations were analyzed using the CAMOIP web server (v.1.1, https://www.camoip.net (accessed on 14 June 2022)) [17], which allows for performing a mutational landscape analysis based on gene expression levels. In this web server, “*ERFE*” was entered into the “Gene Expression” part of the “Mutational Landscape” module for each cancer type in TCGA. Both of the “Driver Mutation” and “Adjust *p*-Value” were chosen to calculate the significance of mutational distribution difference between the *ERFE^high^* and *ERFE^low^* patients. An adjusted *p*-value was calculated by the Benjamini and Hochberg (BH) method using the Fisher’s exact test. To compare the TMB between the *ERFE^high^* and *ERFE^low^* groups, we used the “Tumor Mutation Burden” part of the “Immunogenicity” module for each TCGA tumor dataset. “ERFE” was entered into the “Gene Expression” module to group patients. 

### 4.4. ERFE-Related Gene Enrichment Analysis

SGDA was first performed using the DESeq2 package. We used the median Log_2_ (TPM + 1) value of *ERFE* expression to divide patients into *ERFE^high^* and *ERFE^low^* groups and obtained the relevant DEGs after SGDA. For the GSEA of each tumor, we first filtered the DEGs using adjusted *p*-value < 0.05. Next, the GSEA was performed using the clusterProfiler package (v.3.14.3) [59,60] for the dataset “c2.cp.v7.2.symbols.gmt” obtained from the Molecular Signature Database Collections (https://www.gsea-msigdb.org/gsea/msigdb/index.jsp (accessed on 20 June 2022)) as a reference gene set. The potential *ERFE*-associated functions were inferred as statistically significantly enriched based on a false discovery rate < 0.25 and an adjusted *p*-value < 0.05. Normalized enrichment score (NES) was calculated for each enriched signaling pathway. NES > 0 indicated an enrichment of upregulated pathways associated with the *ERFE^high^* status. NES < 0 indicated an enrichment of downregulated pathways. DEGs were also analyzed by the IPA software (Ingenuity Systems, Redwood City, CA, USA). We filtered the DEGs based on cut-offs of ±2 and <0.05 for fold change and adjusted *p*-values, respectively, followed by core analyses. Initially, each tumor cohort was analyzed using default parameters for predicting canonical pathways associated with the *ERFE^high^* status. Pathways with Z-score ≥ 1 were considered activated upon the *ERFE^high^* level while pathways with Z-score ≤ −1 were considered inhibited. To obtain significant *ERFE*-associated canonical pathways, the list of pathways was further trimmed at *p*-value < 0.05. After obtaining significant *ERFE*-related pathways from the GSEA and IPA, and removing the pathways involving non-tumor diseases, we summarized the upregulated pathways covering at least nine types of tumors.

The ssGSEA is a rank-based algorithm that calculates a score illustrating the level of absolute enrichment of a particular gene set in each sample. We collected the gene sets contained in relevant pathways [61] and introduced them into the ssGSEA for calculating the enrichment score of each sample in each pathway. As an execution tool, R Bioconductor package “Gene Set Variation Analysis” (GSVA, v.3.15) was used with the parameter = “ssgsea”. The output for each signature was a near-Gaussian list of decimals that was used in the visualization/statistical analysis without further processing. The ssGSEA was performed to calculate pathway scores of “Collagen formation” and “Degradation of ECM”.

### 4.5. Immunoscore Assessment

The immune infiltration abundance of each immune cell type was initially calculated by ssGSEA in the investigated 33 tumor types. The Spearman’s rank correlation test was carried out to identify the abundance of immune cells based on *ERFE* expression. Unsupervised clustering of spearman r-values was performed using the Euclidean distance metric with complete linkage. We also used the ESTIMATE package (v. 1.0.13) to calculate the infiltration abundance of stroma cells (stroma score). A potential ICB response was predicted with the TIDE algorithm [40]. The TIDE score was compared between the *ERFE^low^* and *ERFE^high^* statuses using the Mann Whitney U test (Wilcoxon rank sum test). A higher TIDE score indicated potential poor response to ICB therapy. A predicted response rate of ICB treatment from the TIDE analysis was compared between *ERFE^low^* and *ERFE^high^* using the Chi-square test (patient cohort n > 40).

### 4.6. Statistical Analysis

Except for the statistical methods specifically mentioned, all statistical analyses and algorithms were implemented by R software (v. 4.0.3, Vienna, Austria). The ggplot2 package was used to plot or visualize the data. If not stated otherwise, two-group data were performed by the Wilcoxon rank sum test. The Spearman’s rank correlation method was conducted to identify significant abundance relationships. The response rate of ICB treatment was compared between *ERFE^low^* and *ERFE^high^* using the Fisher’s exact test (patient cohort n ≤ 40). *P*-values less than 0.05 were considered statistically significant.

## Data Availability

The data presented in this study are openly available in the TCGA, GTEx, and CCLE projects [47,48,49,50]. The original contributions presented in the study are included in the article/Supplementary Materials. Further inquiries can be directed to the corresponding author/s.

## Supplementary Materials

The following supporting information can be downloaded at: https://www.mdpi.com/article/10.3390/ijms24021725/s1.
