# Supplementary material for: In Silico Pan-Cancer Analysis Reveals Prognostic Role of the Erythroferrone (ERFE) Gene in Human Malignancies
Source: Int J Mol Sci. 2023 Jan 15;24(2):1725. doi: 10.3390/ijms24021725 (PMC9864255; doi:10.3390/ijms24021725)
Supplement: Supplementary file 1 [file ijms-24-01725-s001.zip › ijms-2113255-supplementary.pdf]

Table S1: Multivariable analyses for prognostic significance of *ERFE* expression levels

	Factors in multivariable analyses	HR	95% CI	P-value
Adrenocortical carcinoma **OS**	[a]Clinical T stage, T3&T4 vs. T1&T2	7.744	2.371-25.291	<0.001
	[a]Clinical N stage, N1 vs. N0	2.234	0.618-8.075	0.220
	[a]Clinical M stage, M1 vs. M0	0.889	0.310-2.549	0.826
	Radiation therapy yes vs. no	0.512	0.182-1.437	0.204
	Mitotane therapy yes vs. no	1.400	0.525-3.734	0.501
	***ERFE*, high vs. low expression**	**5.928**	**1.914-18.357**	**0.002**
Adrenocortical carcinoma **DSS**	Clinical T stage, T3&T4 vs. T1&T2	7.744	2.371-25.291	<0.001
	Clinical N stage, N1 vs. N0	2.234	0.618-8.075	0.220
	Clinical M stage, M1 vs. M0	0.889	0.310-2.549	0.826
	Radiation therapy yes vs. no	0.512	0.182-1.437	0.204
	Mitotane therapy yes vs. no	1.400	0.525-3.734	0.501
	***ERFE*, high vs. low expression**	**5.928**	**1.914-18.357**	**0.002**
Adrenocortical carcinoma **PFI**	Clinical T stage, T3&T4 vs. T1&T2	2.547	1.082-5.994	0.032
	Clinical N stage, N1 vs. N0	2.237	0.873-5.734	0.094
	Clinical M stage, M1 vs. M0	1.301	0.509-3.330	0.583
	Radiation therapy yes vs. no	0.641	0.289-1.423	0.275
	Mitotane therapy yes vs. no	1.536	0.723-3.26	0.264
	***ERFE*, high vs. low expression**	**3.336**	**1.607-6.927**	**0.001**
Uveal melanoma **OS**	Clinical T stage, T4 vs. T2&T3	2.544	0.999-6.480	0.050
	***ERFE*, high vs. low expression**	**2.885**	**1.150-7.239**	**0.024**
Uveal melanoma **DSS**	Clinical T stage, T4 vs. T2&T3	3.264	1.165-9.149	0.024
	***ERFE*, high vs. low expression**	**3.080**	**1.154-8.220**	**0.025**
Mesothelioma **OS**	Clinical T stage, T3&T4 vs. T1&T2	0.680	0.371-1.250	0.214
	Clinical N stage, N2&N3 vs. N0&N1	0.909	0.476-1.735	0.772
	Clinical M stage, M1 vs. M0	2.619	0.591-11.601	0.205
	Radiation therapy yes vs. no	0.652	0.358-1.187	0.162
	***ERFE*, high vs. low expression**	**3.177**	**1.640-6.153**	**<0.001**
Mesothelioma **DSS**	Clinical T stage, T3&T4 vs. T1&T2	0.869	0.429-1.761	0.697
	Clinical N stage, N2&N3 vs. N0&N1	0.719	0.339-1.525	0.390
	Clinical M stage, M1 vs. M0	2.947	0.647-13.411	0.162
	Radiation therapy yes vs. no	0.655	0.334-1.286	0.219
	***ERFE*, high vs. low expression**	**2.375**	**1.130-4.993**	**0.022**
Mesothelioma **PFI**	Clinical T stage, T3&T4 vs. T1&T2	1.046	0.545-2.007	0.892
	Clinical N stage, N2&N3 vs. N0&N1	0.770	0.380-1.557	0.466
	Clinical M stage, M1 vs. M0	1.573	0.199-12.445	0.668
	Radiation therapy yes vs. no	0.567	0.300-1.070	0.080
	***ERFE*, high vs. low expression**	**2.015**	**1.035-3.921**	**0.039**
Uterine corpus endometrial carcinoma **OS**	Clinical stage, III&IV vs. I&II	3.973	2.320-6.805	<0.001
	Hormones therapy yes vs. no	0.464	0.182-1.181	0.107
	Radiation therapy yes vs. no	0.496	0.285-0.863	0.013
	Surgical approach open vs. minimally invasive	0.680	0.393-1.175	0.167
	***ERFE*, high vs. low expression**	**2.104**	**1.187-3.729**	**0.011**

Uterine corpus endometrial carcinoma **DSS**	Clinical stage, III&IV vs. I&II	6.337	3.173-12.653	<0.001
	Hormones therapy yes vs. no	0.463	0.139-1.540	0.209
	Radiation therapy yes vs. no	0.435	0.215-0.879	0.020
	Surgical approach open vs. minimally invasive	0.644	0.327-1.269	0.204
	***ERFE*, high vs. low expression**	**2.623**	**1.256-5.476**	**0.010**
Pancreatic adenocarcinoma **OS**	Clinical T stage, T3&T4 vs. T1&T2	0.982	0.352-2.741	0.972
	Clinical N stage, N1 vs. N0	1.791	0.739-4.343	0.197
	Clinical M stage, M1 vs. M0	0.433	0.054-3.477	0.431
	Radiation therapy yes vs. no	0.356	0.144-0.880	0.025
	***ERFE*, high vs. low expression**	**2.738**	**1.391-5.390**	**0.004**
Pancreatic adenocarcinoma **DSS**	Clinical T stage, T3&T4 vs. T1&T2	1.325	0.375-4.684	0.662
	Clinical N stage, N1 vs. N0	1.885	0.707-5.026	0.205
	Clinical M stage, M1 vs. M0	0.514	0.063-4.165	0.533
	Radiation therapy yes vs. no	0.355	0.133-0.947	0.039
	***ERFE*, high vs. low expression**	**2.268**	**1.104-4.660**	**0.026**
Pancreatic adenocarcinoma **PFI**	Clinical T stage, T3&T4 vs. T1&T2	1.270	0.517-3.119	0.602
	Clinical N stage, N1 vs. N0	1.270	0.648-2.489	0.487
	Clinical M stage, M1 vs. M0	0.729	0.205-2.591	0.625
	Radiation therapy yes vs. no	1.022	0.537-1.945	0.948
	***ERFE*, high vs. low expression**	**1.915**	**1.083-3.385**	**0.025**
Kidney renal clear cell carcinoma **OS**	Clinical T stage, T3&T4 vs. T1&T2	2.333	1.481-3.675	<0.001
	Clinical N stage, N1 vs. N0	1.755	0.901-3.417	0.098
	Clinical M stage, M1 vs. M0	2.735	1.703-4.390	<0.001
	***ERFE*, high vs. low expression**	**1.600**	**1.049-2.439**	**0.029**
Kidney renal clear cell carcinoma **DSS**	Clinical T stage, T3&T4 vs. T1&T2	3.174	1.720-5.856	<0.001
	Clinical N stage, N1 vs. N0	1.389	0.632-3.052	0.413
	Clinical M stage, M1 vs. M0	4.745	2.716-8.291	<0.001
	***ERFE*, high vs. low expression**	**1.963**	**1.145-3.364**	**0.014**
Kidney renal clear cell carcinoma **PFI**	Clinical T stage, T3&T4 vs. T1&T2	2.649	1.626-4.318	<0.001
	Clinical N stage, N1 vs. N0	1.199	0.587-2.452	0.618
	Clinical M stage, M1 vs. M0	5.750	3.523-9.387	<0.001
	***ERFE*, high vs. low expression**	**1.634**	**1.044-2.558**	**0.032**
Colon adenocarcinoma **OS**	Clinical T stage, T3&T4 vs. T1&T2	3.538	1.093-11.456	0.035
	Clinical N stage, N2 vs. N0&1	1.870	1.140-3.066	0.013
	Clinical M stage, M1 vs. M0	2.961	1.779-4.928	<0.001
	***ERFE*, high vs. low expression**	**1.921**	**1.219-3.027**	**0.005**
Colon adenocarcinoma **DSS**	Clinical T stage, T3&T4 vs. T1&T2	2.769	0.647-11.850	0.170
	Clinical N stage, N2 vs. N0&1	1.542	0.843-2.822	0.160
	Clinical M stage, M1 vs. M0	6.213	3.362-11.482	<0.001
	***ERFE*, high vs. low expression**	**2.555**	**1.420-4.599**	**0.002**
Colon adenocarcinoma **PFI**	Clinical T stage, T3&T4 vs. T1&T2	1.462	0.740-2.889	0.274
	Clinical N stage, N2 vs. N0&1	1.956	1.260-3.036	0.003
	Clinical M stage, M1 vs. M0	4.482	2.884-6.966	<0.001
	***ERFE*, high vs. low expression**	**1.580**	**1.076-2.322**	**0.020**
Skin cutaneous Melanoma **OS**	Clinical T stage, T3&T4 vs. T1&T2	1.947	1.378-2.751	<0.001
	Clinical N stage, N2&N3 vs. N0&N1	2.604	1.753-3.867	<0.001
	Clinical M stage, M1 vs. M0	1.525	0.656-3.547	0.327

	Radiation therapy yes vs. no	1.074	0.719-1.603	0.727
	***ERFE*, high vs. low expression**	**1.582**	**1.145-2.187**	**0.005**
Skin Cutaneous Melanoma **DSS**	Clinical T stage, T3&T4 vs. T1&T2	1.765	1.232-2.529	0.002
	Clinical N stage, N2&N3 vs. N0&N1	2.759	1.819-4.183	<0.001
	Clinical M stage, M1 vs. M0	1.673	0.716-3.910	0.235
	Radiation therapy yes vs. no	1.139	0.750-1.730	0.541
	***ERFE*, high vs. low expression**	**1.566**	**1.112-2.206**	**0.010**
Skin Cutaneous Melanoma **PFI**	Clinical T stage, T3&T4 vs. T1&T2	1.624	1.218-2.165	<0.001
	Clinical N stage, N2&N3 vs. N0&N1	2.229	1.590-3.124	<0.001
	Clinical M stage, M1 vs. M0	1.473	0.739-2.934	0.271
	Radiation therapy yes vs. no	1.251	0.897-1.744	0.186
	***ERFE*, high vs. low expression**	**1.462**	**1.113-1.921**	**0.006**
Cervical squamous cell carcinoma and endocervical adenocarcinoma **PFI**	Clinical stage, III&IV vs. I&II	1.677	0.994-2.829	0.053
	Radiation therapy yes vs. no	1.115	0.640-1.942	0.700
	***ERFE*, high vs. low expression**	**1.653**	**1.020-2.679**	**0.041**
Head and Neck squamous cell carcinoma **OS**	Clinical T stage, T3&T4 vs. T1&T2	1.834	1.270-2.647	0.001
	Clinical N stage, N2&N3 vs. N0&N1	1.507	1.082-2.097	0.015
	Clinical M stage, M1 vs. M0	5.739	1.804-18.263	0.003
	Radiation therapy yes vs. no	0.470	0.339-0.653	<0.001
	***ERFE*, high vs. low expression**	**1.461**	**1.072-1.993**	**0.016**
Kidney renal papillary cell carcinoma **OS**	Clinical T stage, T3&T4 vs. T1&T2	4.228	2.055-8.698	<0.001
	***ERFE*, high vs. low expression**	**0.333**	**0.142-0.781**	**0.011**

[a]The TNM Classification of Malignant Tumors (TNM) was used in the multivariable analyses. T describes the size of the original (primary) tumor and whether it has invaded nearby tissue. N describes nearby (regional) lymph nodes that are involved. M describes distant metastasis.

Abbreviations: OS, overall survival; DSS, Disease−specific survival; PFI, Progression−free interval; T, tumor; N, lymph nodes; M; metastasis.

Table S2: Comparison of response to anti-PD-1 treatment in *ERFE*^{low} and *ERFE*^{high} melanoma in real world

Study	Cancer	Drug	Response in *ERFE*^{low}	Response in *ERFE*^{high}	P-value
Hugo et al. (2016) [36]	Melanoma	Anti-PD-1 (Pembrolizumab, Nivolumab)	69% (9/13)	33% (4/12)	0.1152
Riaz et al. (2017, naïve) [37]	Melanoma	Anti-PD-1 (Nivolumab), no Ipilimumab treatment before Nivolumab therapy	15% (2/13)	33% (4/12)	0.3783
Riaz et al. (2017, progressed) [37]	Melanoma	Anti-PD-1 (Nivolumab), patients progressed on Ipilimumab	31% (4/13)	0% (0/13)	0.0957
Gide et al. (2019, monotherapy) [38]	Melanoma	Anti-PD-1 (Pembrolizumab, Nivolumab)	50% (10/20)	43% (9/21)	0.7579
Gide et al. (2019, combination) [38]	Melanoma	Anti-PD-1 (Pembrolizumab, Nivolumab); Anti-CTLA4 (Ipilimumab)	63% (10/16)	69% (11/16)	>0.9999

Clinical data of the three studies were downloaded from TIDE database [40].

Figure S1

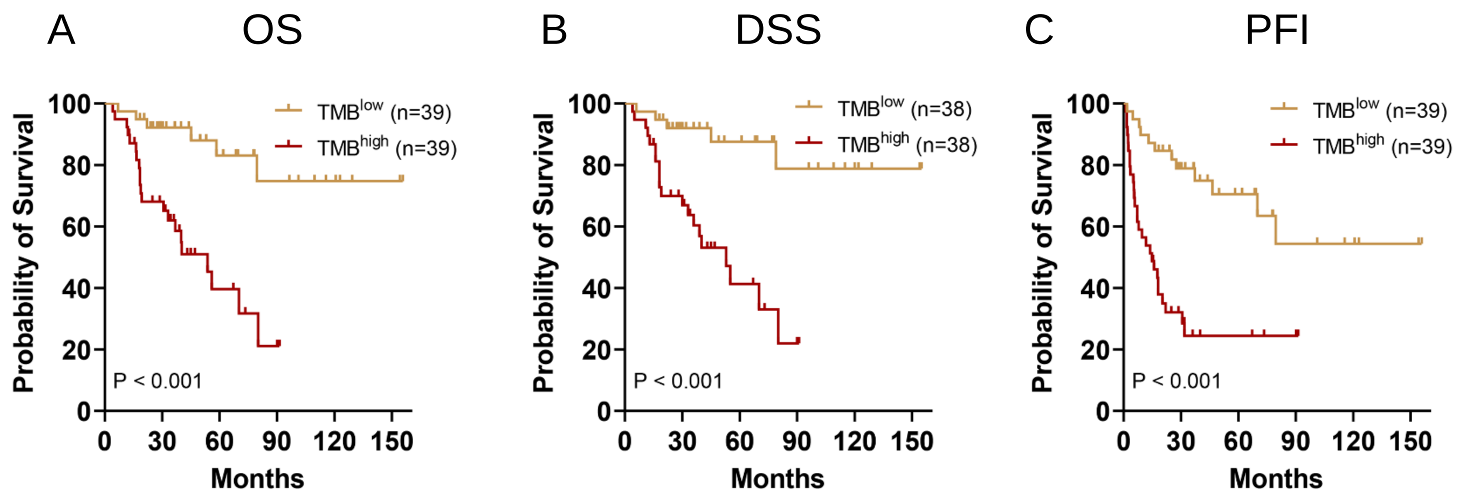

Figure S1. The correlation between TMB and survival outcomes in adrenocortical carcinoma (ACC). Patients were grouped by the median TMB levels. OS (**A**), DSS (**B**) and PFI (**C**) between TMB^{low} and TMB^{high} groups were analyzed by Kaplan–Meier survival analysis via Log-rank test.

Figure S2

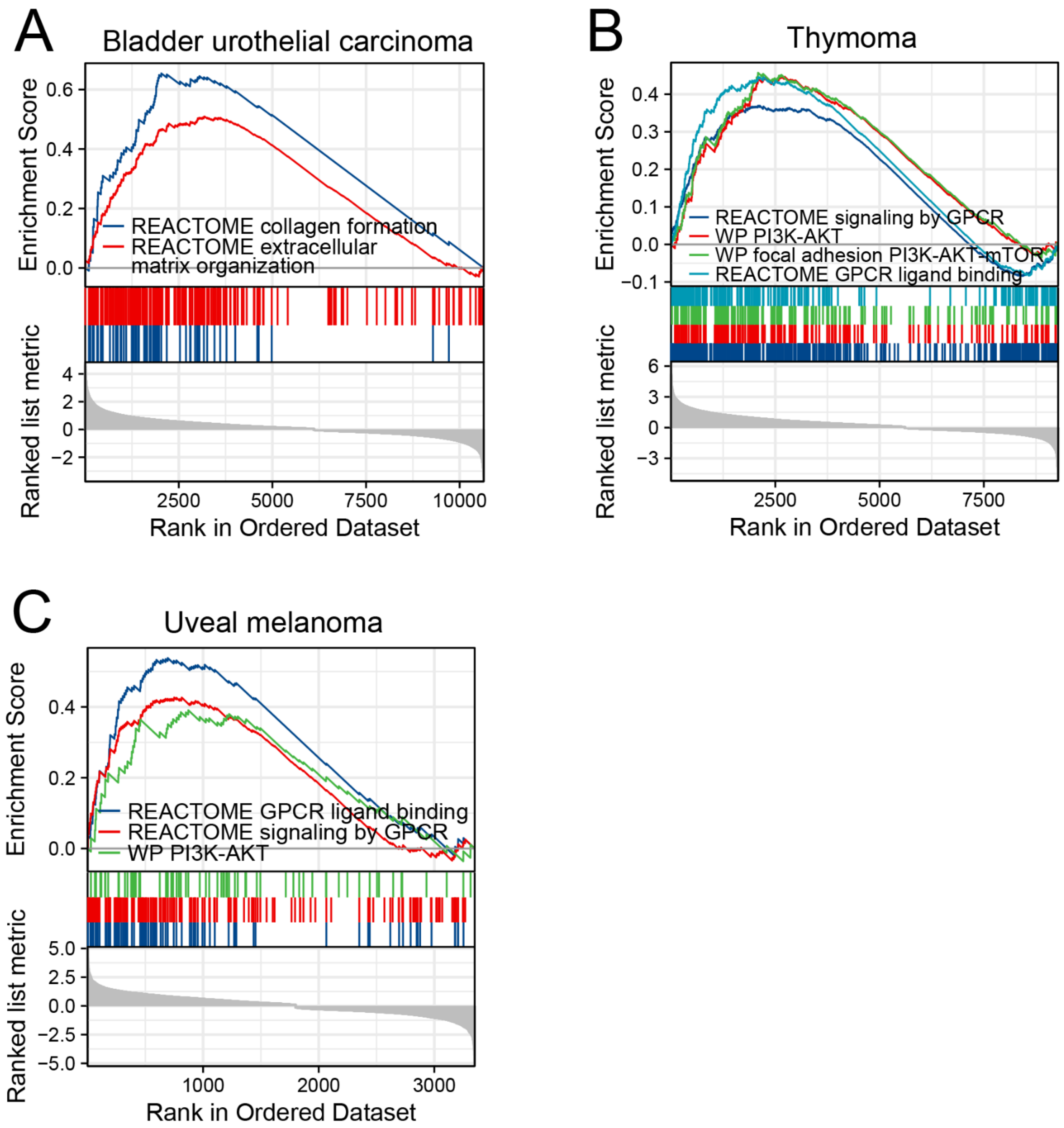

Figure S2. GSEA results are shown for upregulated ECM, GPCR and PI3K-AKT signaling pathways in bladder urothelial carcinoma, thymoma and uveal melanoma upon *ERFE*^{high} expression.

Figure S3

IPA in bladder urothelial carcinoma: the activated tumor microenvironment pathway

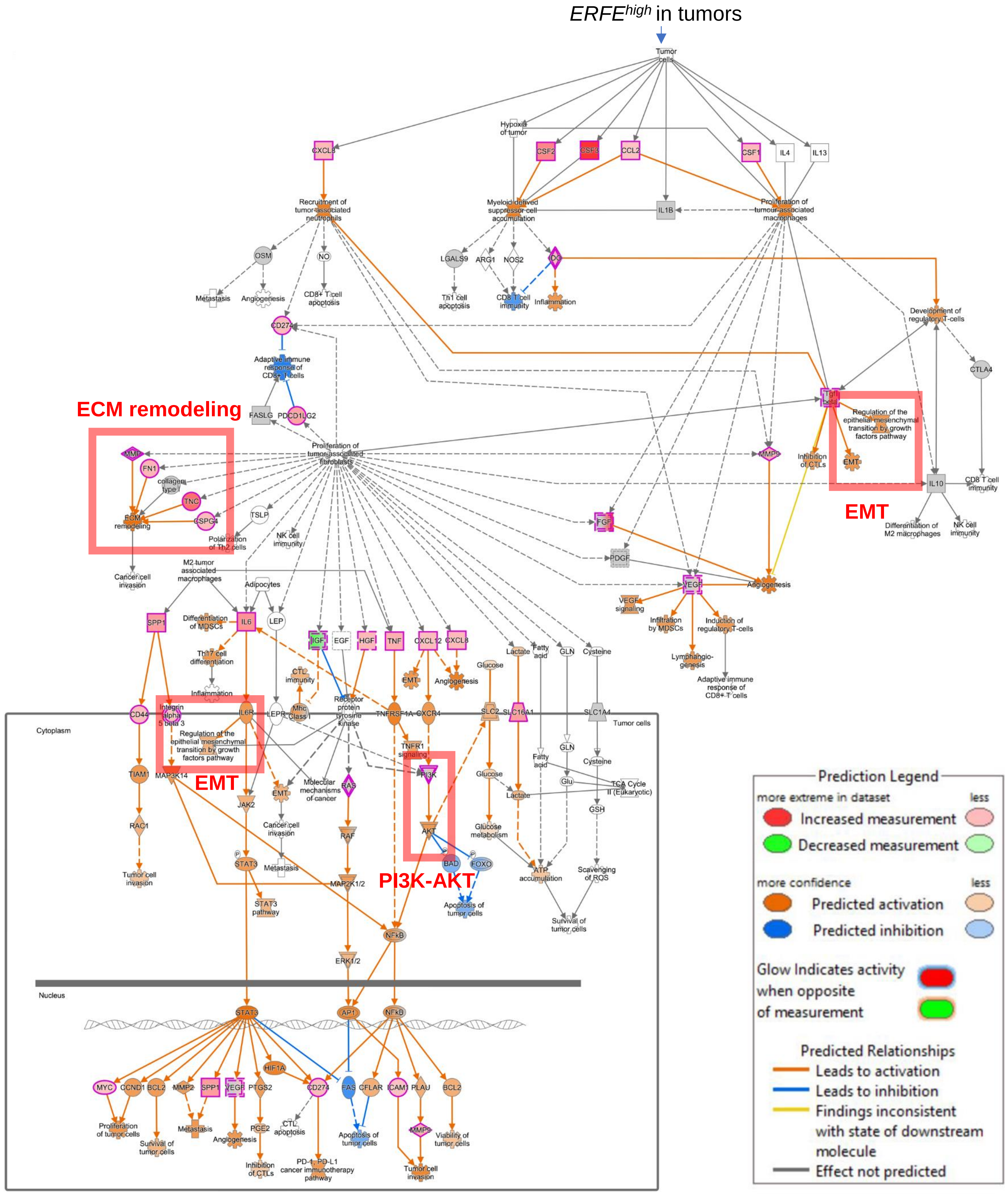

Figure S3. Example of the activated tumor microenvironment pathway upon *ERFE*^{high} status in bladder urothelial carcinoma. Abbreviation: EMT, epithelial-mesenchymal transition.

Figure S4

IPA in thymoma: the activated signaling pathway involved in Breast cancer regulation by Stathmin1 (STMN1)

ERFE^{high} in tumors

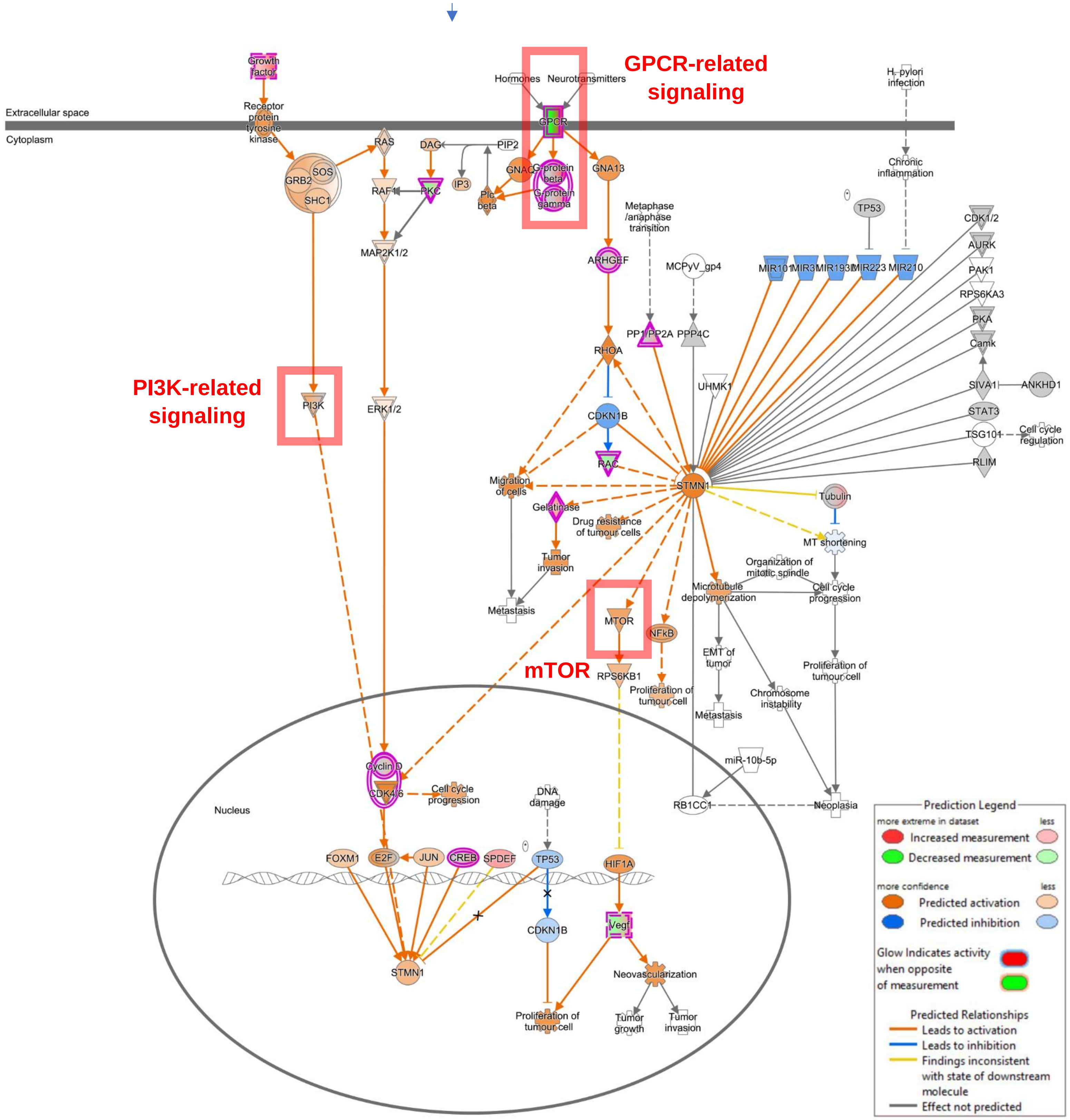

Figure S4. Example of the activated signaling pathway involved in breast cancer regulation by Stathmin1 upon *ERFE*^{high} status in thymoma.

Figure S5

IPA in uveal melanoma: the activated G-protein coupled receptor (GPCR) signaling pathway

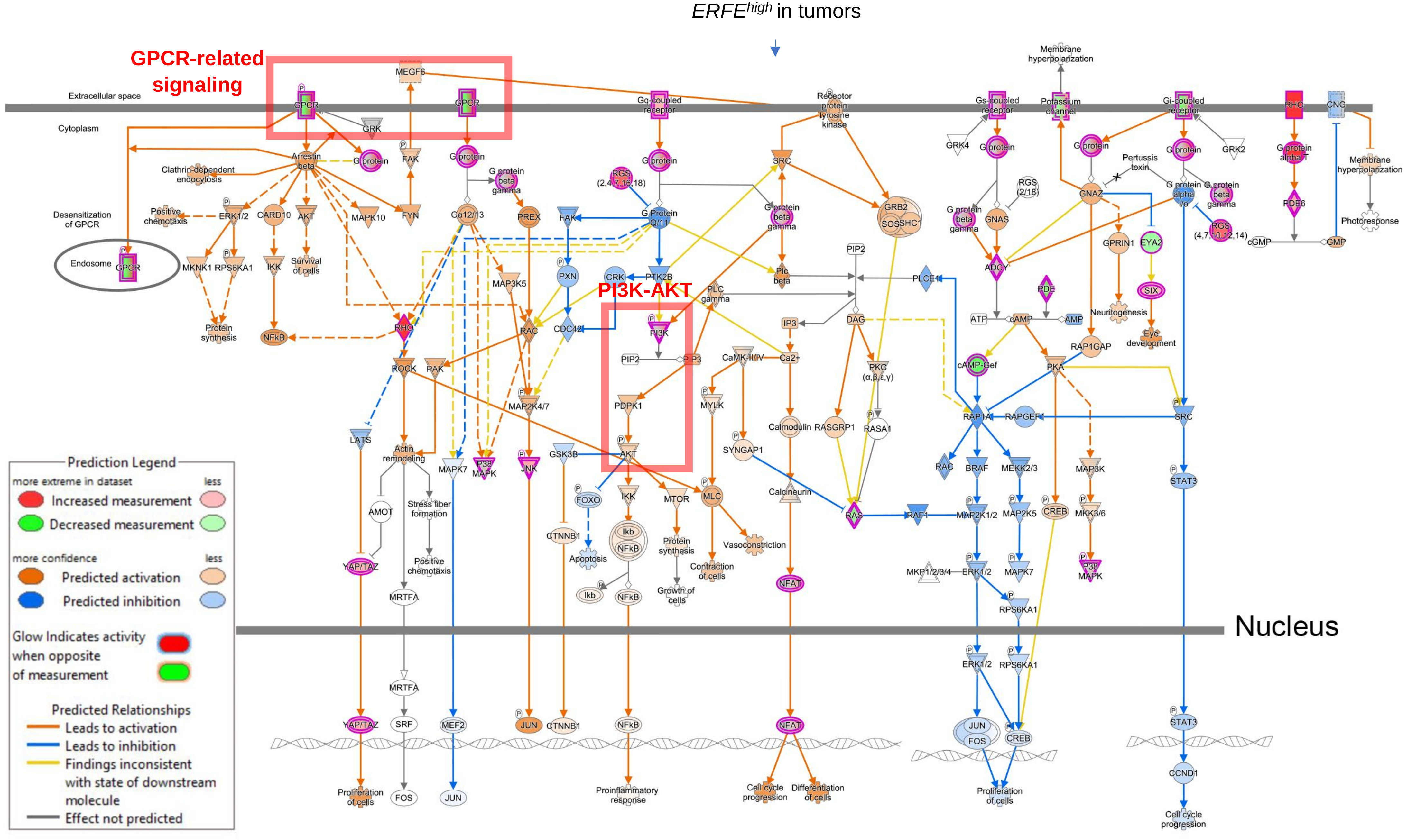

Figure S5. Example of the activated GPCR signaling pathway upon *ERFE*high status in uveal melanoma.

Figure S6

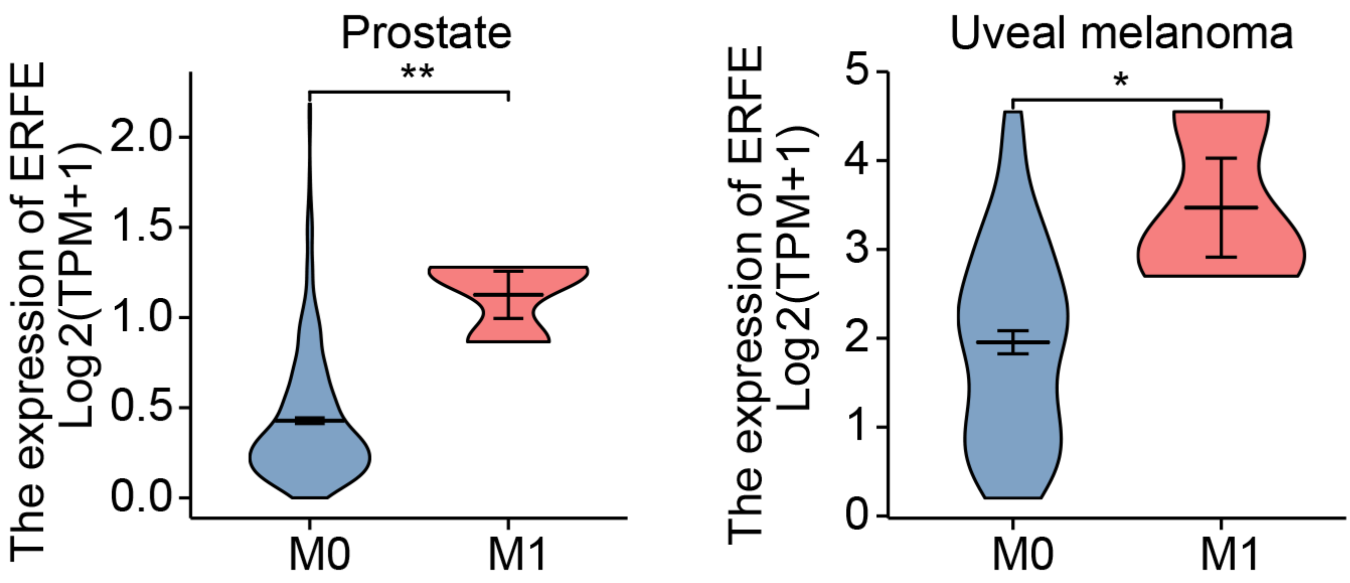

Figure S6. *ERFE* expression was compared between different distant metastasis status (M0 vs. M1) in prostate cancer and uveal melanoma. M0 indicates no distant metastasis. M1 indicates the occurrence of distant metastasis. The statistical difference of the two groups was compared by Wilcoxon rank sum test. The data are median±interquartile range (IQR). Asterisks (*) stand for significance levels. *$p < 0.05$; **$p < 0.01$.

Figure S7

Adrenocortical carcinoma: IL-17 signaling pathway

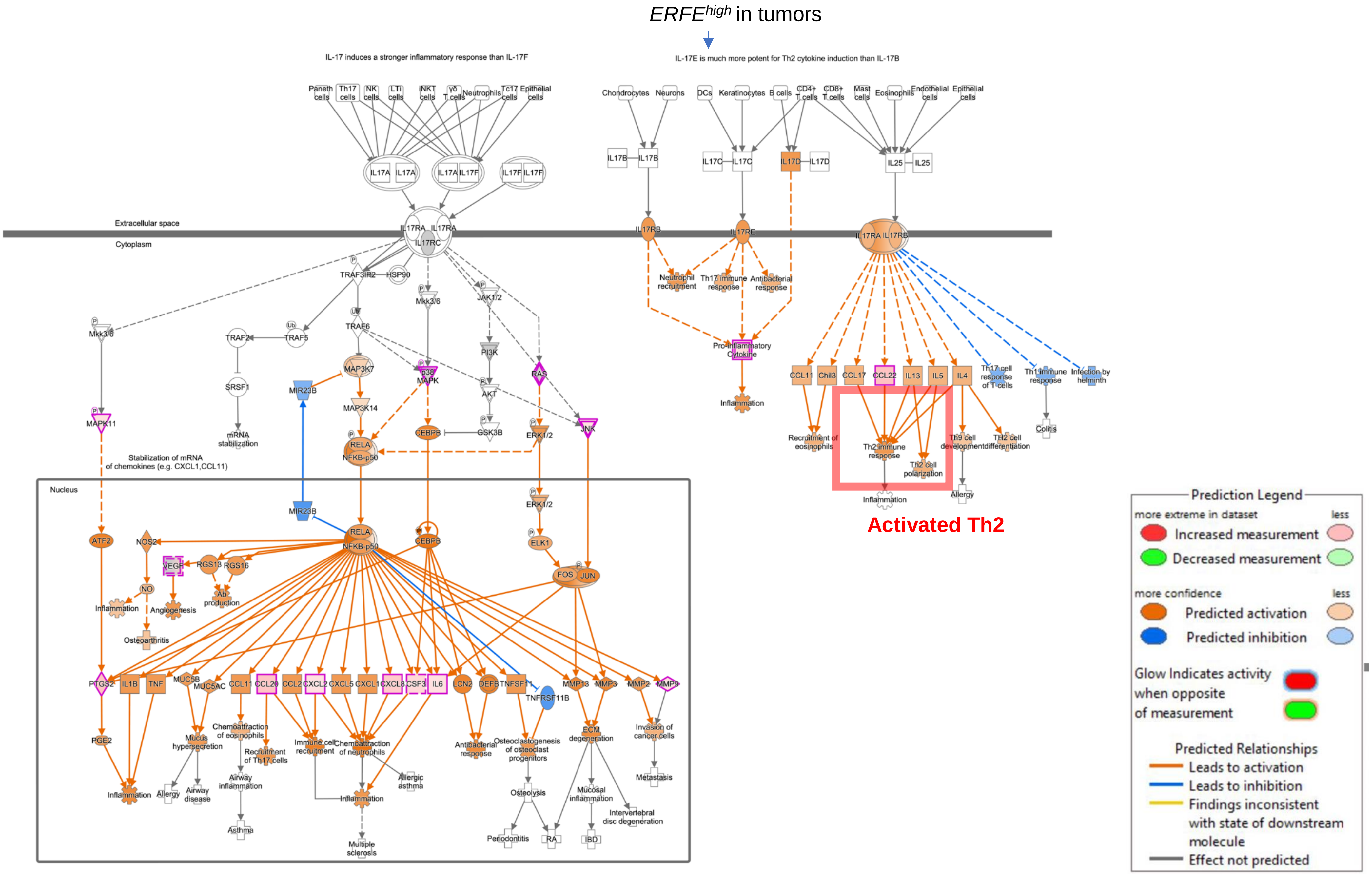

Figure S7. Example of the activated IL-17 signaling pathway upon *ERFE*^{high} status in adrenocortical carcinoma.

Figure S8

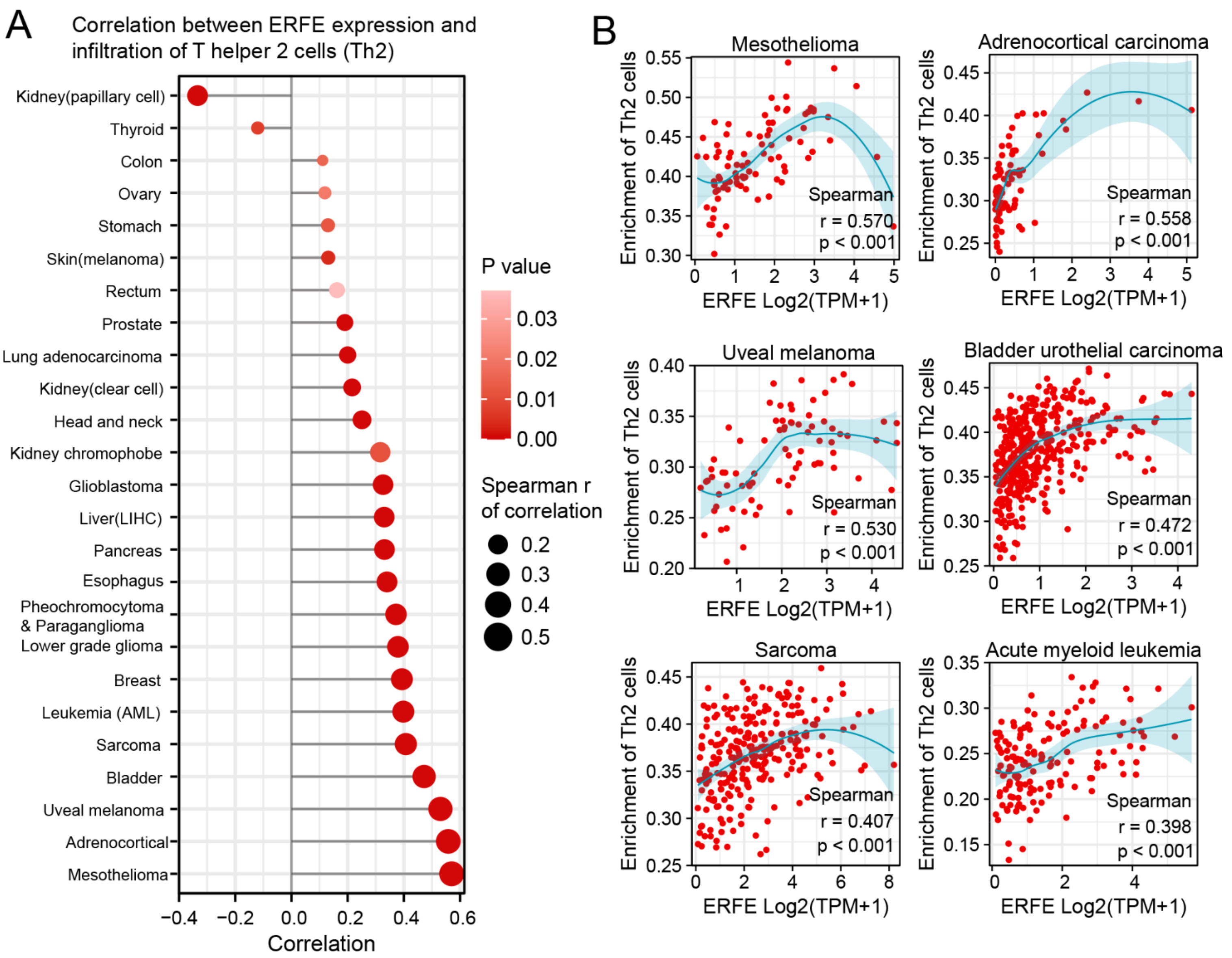

Figure S8. Correlation of *ERFE* expression with T helper 2 (Th2) immune infiltrate in pan cancer. (**A**) Relationships between Th2 cell infiltration and *ERFE* expression in pan cancer are shown. Th2 cell infiltration score was calculated by ssGSEA. (**B**) Examples of correlation between *ERFE* expression and Th2 infiltration are shown in 6 types of tumors (Spearman r > 0.350, p < 0.001). Spearman's rank correlation test was carried out. Results with statistical significance (p < 0.05) are displayed.

Figure S9

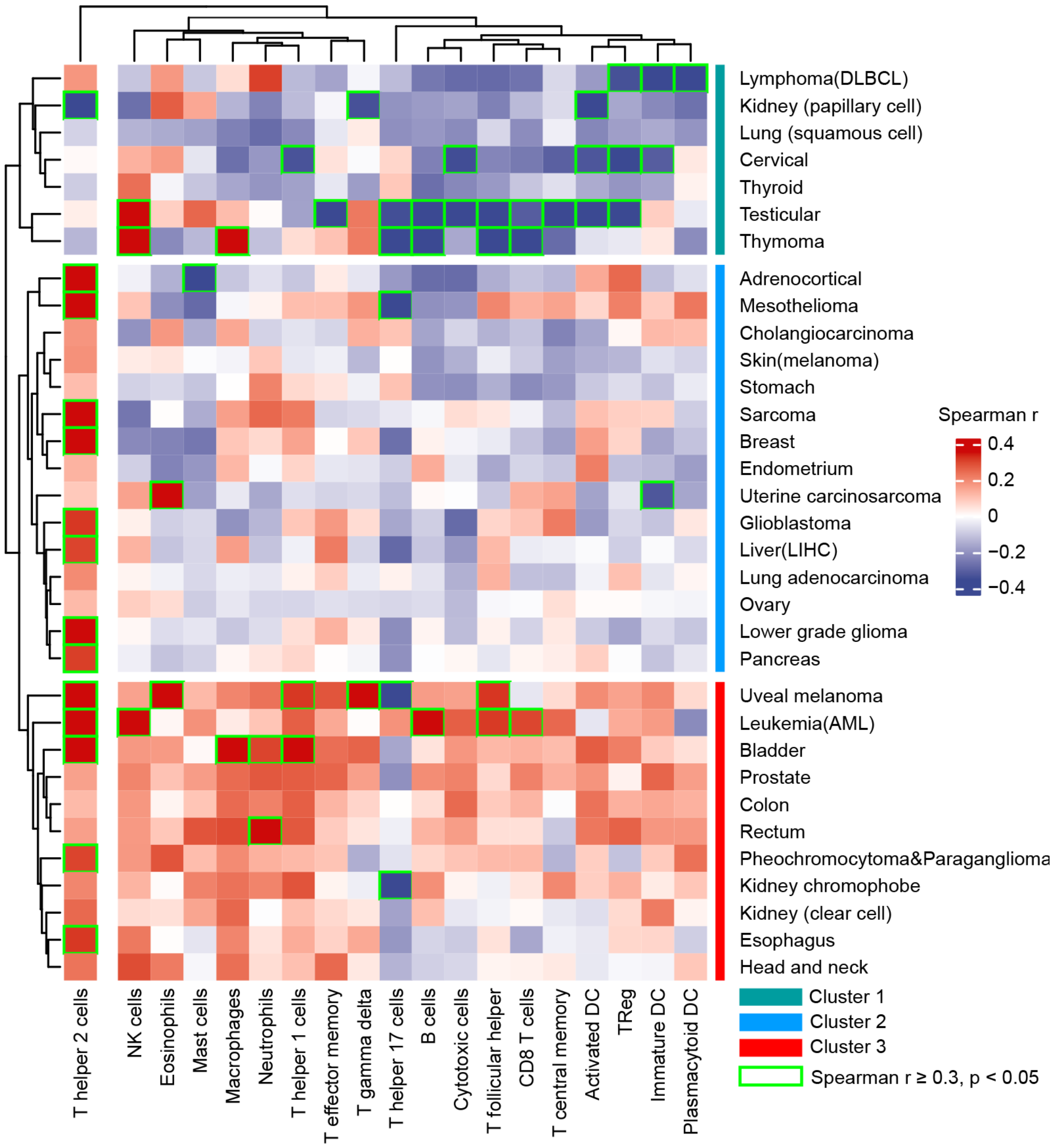

Figure S9. Heatmap showing spearman correlation coefficient between *ERFE* expression and the fraction of infiltrated immune cells in pan cancer. Immune cell infiltration score was firstly calculated by ssGSEA. Spearman's rank correlation test was carried out. Red color indicates positive correlation, blue color indicates negative correlation. Unsupervised clustering used Euclidean distance metric with complete linkage.

Figure S10

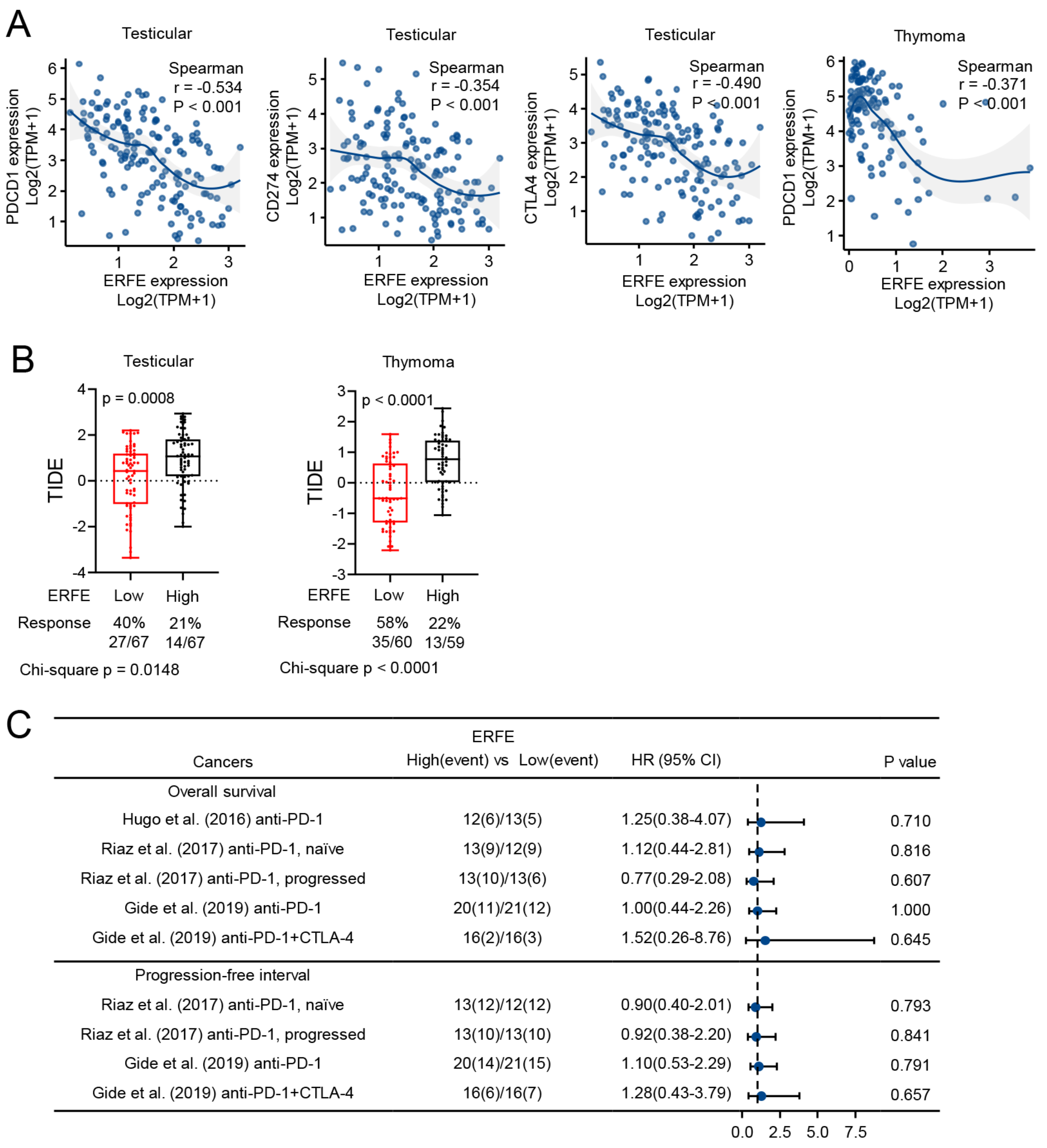

Figure S10. Association of *ERFE* expression status with response to immune checkpoint blockade (ICB) therapy. (**A**) Association of *ERFE* expression with immune checkpoint gene expression (*PDCD1*, *CD274* and *CTLA4*) in testicular cancer and thymoma. Spearman's rank correlation test was carried out. (**B**) Potential ICB response was predicted using TIDE algorithm and compared between $ERFE^{low}$ and $ERFE^{high}$ status in testicular cancer and thymoma. TIDE score was compared using Mann Whitney U test. Higher TIDE score indicates potential poor response to ICB therapy. Predicted response rate of ICB treatment was compared between $ERFE^{low}$ and $ERFE^{high}$ status using Chi-square test. (**C**) Forest plot of survival (OS and PFI) associations with *ERFE* expression levels in patients with melanoma receiving anti-PD-1 treatment. Log-rank test was conducted.
